# The Influence of Creative Personality and Goal Orientation on Innovation Performance

**DOI:** 10.3389/fpsyg.2021.634951

**Published:** 2021-04-09

**Authors:** Keqiucheng Zhou

**Affiliations:** School of Psychological and Cognitive Sciences, Peking University, Beijing, China

**Keywords:** creative personality, employee creativity performance, goal orientation, innovation performance, learning goal orientation

## Abstract

The complexity and challenges of the external environment accelerate the awakening of the new generation of enterprise employees’ self-consciousness. Facing the continuous expansion of the information-based work mode, the traditional management mechanism of enterprises has a more limited impact on employee performance. Based on the goal-oriented theory, developing and excavating the creative personality traits of employees, making full use of goal-oriented behavior to improve their own innovation performance management path, are expected to become a new path to continuously enhance the innovation ability of enterprises. In this study, we take the employees of high-tech enterprises as samples to explore the influence mechanism of creative personality traits, goal orientation and employee innovation performance. The results show that goal orientation significantly moderates the relationship between creative personality traits and innovation performance. The mediating effects of learning goal orientation, performance certification orientation, and performance avoidance orientation are all significant.

## Introduction

At present, international competition is becoming more fierce. On the one hand, environment that enterprises face is changing with each passing day, innovation has become one of the key skills for enterprises to cope with the changes in the external environment (Zhang et al., 2014). On the other hand, in terms of management practices, enterprises are facing an increasingly complex environment. The new generation of enterprise employees’ self-consciousness gradually awakens, and the changes of external factors, such as the emergence of information-based work model, are constantly blurring the boundaries of traditional management and challenging the traditional leadership model, thus prompting enterprises to urgently need employees to show high initiative, spontaneity and self-management skills (Hu, 2017).

Therefore, both the development of theoretical constructs and the demands of management practice indicate that we should re-examine the employee innovation mechanism from the perspective of employees’ spontaneous, active planning and management self-behavior. Based on the goal-oriented theory, it can provide a theoretical basis for the study of how employees actively manage and promote their own innovation. The theory focuses on the initiative and spontaneity of employees in their work and the goal orientation of employees’ innovation (Crant, 2000; Lukasz, 2019). The goal orientation is divided into three categories: learning goal orientation, performance certification orientation, and performance avoidance orientation (Vande, 1997), which determines the behavior motivation of employees, and has an important impact on the innovation process and innovation results. Specifically, employees will actively seek information, opportunities, improve the *status quo*, and manage their own behavior to complete the work (Parker et al., 2010). Bateman and Crant (1993) pointed out that goal oriented individuals are good at seeking new information and practice in order to achieve their own goals or improve their performance. At the same time, goal oriented employees are better at identifying opportunities and often strive to accomplish tasks beyond their role expectations (Bateman and Crant, 1993). Crant (2000) points out that goal oriented employees will seek feedback more actively, frequently, and actively to improve their performance. Therefore, we should not neglect the impact of internal factors, creative personality traits and goal orientation, on employee innovation performance.

Employee innovation performance is a product, process or method that is new, feasible, and valuable to the organization at the individual level (Shalley et al., 2004). How to effectively improve employee innovation performance is an important issue of common concern to researchers and managers. Most of the existing studies focus on the individual and environmental factors that affect employees’ innovation performance. Such as creative personality traits, learning orientation of employees (Luisa et al., 2014), as well as resources provided by the organization, time pressure, evaluation and reward system, leadership style, innovation atmosphere, work characteristics, and so on (Itaya, 2014). It is worth noting that many studies only focus on the single factor of employee innovation performance, such as focus on the impact of goal orientation, leadership style, work characteristics, and innovation atmosphere on employee innovation performance. Therefore, this paper believes that based on the creative personality traits of employees, from the goal-oriented perspective to explore the issue of employee innovation performance, will further expand and deepen the existing research.

## Theory and Hypothesis

### Theoretical Background

Creative personality profile, first proposed and used by Guilford (Guilford, 2003), refers to personality traits related to creative activity and achievement (Danilo, 2019). Although different scholars have different definitions of creative personality traits, there are some widely accepted viewpoints. For example, people with creative personality traits tend to have a wide range of interests and tend to be exposed to diverse information and perspectives, thus contributing to the development of new approaches to problems (Barron and Harrington, 1981). In addition, creative personality traits such as initiative, independent judgment, openness, persistence, and willingness to take risks are also considered to help individuals form creative ideas (Mumford, 2012).

Creative personality trait in complex social environment is an important factor to explain innovation performance (Woodman et al., 1993). Creative personality traits are the dynamic system of individual innovation and the personality characteristics related to creativity. These characteristics promote the individual to strengthen creative consciousness and produce innovative behavior. Although most enterprises hope to know the result of creative behavior, there is still a long way to go from the creation of ideas to the achievement of innovative results.

According to the trait theory, creativity, as a relatively independent ability, derives largely from innate potential, which makes individuals willing to break the risk in the environment (Vervalin, 1962). In face of various obstacles, there are still some individuals in the organization who put forward and practice their own creative ideas, thus showing innovative behavior. This part of the individual in the practice of their creative ideas, often do not define this behavior as innovation or creativity. They just think that they are doing what is right or meaningful to themselves, this is the performance of creative personality traits (Meyerson and Scully, 1995). It is further demonstrated the existence of creative personality traits as a specific personality trait, and proposed the intrinsic driving mechanism of creative personality traits (Schoen et al., 2016). The Individuals who have the creative personality traits, will show more innovative than other individuals get higher innovation to performance (Gu et al., 2016).

Goal orientation refers to the active role-oriented temperament of individuals actively initiating changes and striving to influence the environment (Bateman and Crant, 1993). Individual innovation performance is influenced by many factors including personality (Tierney et al., 1999). The goal-oriented approach is helpful to better understand the behavior patterns of individual work (Ford, 1996). According to the motivated action theory, specific personality traits promote individuals to establish corresponding goal orientation, which leads to different behaviors (DeShon and Gillespie, 2005). In addition, in achievement motivation theory, goal orientation is closely related to intrinsic motivation (Heyman and Dweck, 1992). Goal-oriented theory holds that employees play an active role in their work behavior, they will actively create an environment, improve conditions, and actively seek opportunities and information instead of passively waiting to be managed (Crant, 2000; Li et al., 2010; Parker et al., 2010).

There are three perspectives on the definition of goal orientation in the existing research: First, personality traits perspective. From the perspective of personality traits, Bateman, and others believe that goal orientation, as a stable personality trait, can distinguish the degree to which people take action to influence and improve their environment. Employees with goal-oriented personality traits are not willing to be constrained by environmental factors, and it have a strong desire to change the environment, and are better at actively completing their work (Bateman and Crant, 1993). Second, from the perspective of behavior, goal orientation is a spontaneous and active behavior pattern, corresponding to negative and reactive behavior, to improve the environment and thus to improve one’s own situation (Parker et al., 2010). Third, process perspective. Goal orientation is a dynamic process. It includes individual expectations, preparation and future-oriented actions. From the perspective of process, individuals ultimately influence the future through the production of corresponding goal orientation (Grant and Ashford, 2008). The goal-oriented process view can accurately capture the impact of goal orientation, especially the impact of goal orientation on individual performance (Crant, 2000). Based on this, this paper combines goal-oriented and creative personality trait research, we will analyze the internal relationship among creative personality traits, goal orientation and innovation performance.

### Hypothesis

#### Goal Orientation and Innovation Performance

The concept of Goal orientation embodies the inherent tendency of individuals to commit themselves to constructive change. Compared with those passively adapting to the environment, goal-oriented individuals are good at identifying opportunities, taking action and persisting until their goals are achieved (Crant, 2000). Previous studies have shown that goal orientation is related to many workplace behaviors, such as job performance, organizational citizenship behavior, and so on (Li et al., 2010). Goal orientation can affect employee innovation performance through the following three paths:

First of all, the goal-oriented individuals are good at seeking new information and practice, in order to achieve their own goals or improve their own performance (Bateman and Crant, 1993). Goal-oriented employees are better at identifying opportunities and often try to do more than their role expected. The goal-oriented employees are more active, frequent, and proactive in seeking feedback to improve their performance (Crant, 2000).

Secondly, the goal-oriented employees show more behavior and initiative than the expectation of ordinary job requirements, which also provide the motivation for their initiative to improve innovation performance. Such employees tend to proactively update their professional knowledge and work skills, etc., which provide them with a knowledge base for showing high innovation performance. In addition, goal-oriented employees show behaviors that exceed the expectations of ordinary work requirements and initiative, which also provide a motivational basis for actively improving innovation performance. These knowledge skills and innovation motives are the two powerful driving forces for employees to abandon their habitual behaviors and adopt innovations (Unsworth and Clegg, 2010).

Thirdly, the goal-oriented individuals have stronger social network building ability and social capital (Thompson, 2005). Individual innovation process has certain sociality, more social network ties an individual has, the more diverse information and views it gets, and more resources and opportunities would be used to carry out active innovation and change (Ohly et al., 2010). In the empirical study, the goal orientation and individual innovation performance have a positive relationship through meta-analysis (Fuller and Marler, 2009). The goal orientation plays an important role in the creation of creativity, and points out that the importance of goal orientation lies in the difference between “what can be done” and “what should be done.” If employees have a higher goal orientation, they focus on their work (Amabile, 1983). If employees are highly goal-oriented, they will concentrate on their work (Deci and Ryan, 2004). For example, the performance-oriented employees will be driven by high intrinsic motivation to work in the task, while enjoying the work process, and strive to find better new solutions, in order to make results to prove their ability (Swift et al., 2010). If an employee is not interested in a task, even with a high learning goal orientation, he or she may be reluctant to work hard to learn new knowledge about the task, making it difficult to find useful new solutions. Similarly, if employees do not focus on their work, they do not put their energy into creative processes that solve difficult work problems, making it difficult to improve employee innovation performance. Therefore, we hypothesis that the three dimensions of goal orientation (learning goal orientation, performance certification orientation, and performance avoidance orientation) affect employees’ innovation performance.

Hypothesis 1:

H1a: Learning goal orientation has a positive impact on innovation performance.
H1b: Performance certification orientation is positively related to employee innovation performance, while performance avoidance orientation is negatively related to employee innovation performance.

#### Creative Personality Traits and Goal Orientation of Employees

Everyone has different creative personality traits (Guo and Duan, 2008). Creative personality traits are positively related to the number of patents of R & D personnel, which is one of the objective indicators of creativity (Ferguson, 2010). Because behavior is a function of an individual and his environment, the interaction between personality traits and the environment should be examined in order to accurately examine the influence of creative personality traits. Trait activation theory provides theoretical support for explaining the interaction between individual personality traits and environmental dynamics. The human-situation interaction model in the theory lays a foundation for explaining the conditions of predicting job performance and personal behavior by personality traits (Wang and Sun, 2012). According to the theory, trait activation is a process in which an individual presents characteristics in trait-related contexts, and the relationship between individual traits and behavior outcomes is stronger in contexts suitable for trait expression (Tett and Burnett, 2003). Meanwhile, in the study of the moderating effect of personality traits in different contexts, it is considered that under the different intensity of time pressure, the moderating effect of personality traits on environmental support and creative performance of employees was discussed (Baer and Oldham, 2006).

Hypothesis 2:

H2a: Creative personality traits have a positive impact on employees’ innovation performance.

#### The Effect of Goal Orientation on the Relationship Between Employees’ Creative Personality Traits and Innovation Performance

Goal orientation refers to the individual’s pursuit of a goal in an achievement situation, which affects how the individual understands, deals with, and responds to the achievement situation. Goal orientation can often predict individual behavior and performance (Elliot and Thrash, 2002). Employees’ innovation performance often results from goal-oriented behavior. Employees’ different goal orientations help explain the differences in their creativity at work.

As one of the goal orientations, learning goal orientations emphasize the acquisition of knowledge, the improvement of self-ability and the mastery of tasks. The performance certification orientation emphasizes proving one’s own ability and obtaining praise from others, while the performance avoidance orientation emphasizes avoiding negative evaluation of one’s own ability. Individuals with performance avoidance orientation are often reluctant to face challenging work to avoid risks. Creative personality includes taking risks and sticking to challenging work. Therefore, in this paper, the research content will include the learning goal-oriented, performance certification-oriented, and performance avoidance-oriented.

Goal orientation is related to specific personality traits to some extent, and personality traits influence individual behavior through goal orientation (DeShon and Gillespie, 2005). Individuals with creative personality traits are willing to challenge complex tasks and therefore have greater enthusiasm for new knowledge and skills and seek breakthroughs through continuous learning. Individuals with this personality trait are more confident that intelligence can be acquired through learning and that learning goal orientation is easier to develop.

In addition, individuals with creative personality traits are aggressive, tend to prefer work that affects others, and strive to gain recognition from work and prove their ability (Benjamin et al., 2018). Therefore, creative personality traits are closely related to learning goal orientation and performance proof goal orientation. Goal-oriented individuals invest more effort in mastering new knowledge and skills, which are the foundation of creativity, when faced with difficulties (Hirst et al., 2009).

Learning goal-oriented individuals tend to be more responsible, and through learning to enhance confidence in their work, to have the courage to break through and seek better new methods (Lu and Chang, 2007). When individuals have performance certification orientation, they enhance their prestige by sharing knowledge and information with their superiors or central people in the network (Swift et al., 2010), which is helpful for the dissemination of creative ideas and plays an important role in creativity (Armbrecht et al., 2001). In addition, individuals with performance certification orientation have higher achievement motivation and will actively improve their work and find better new methods (Zhou et al., 2008). We therefore propose that:

Hypothesis 3:

H3a: Goal orientation moderates the relationship between creative personality and employee innovation performance.
H3b: Goal orientation has a mediating effect between creative personality traits and innovation performance.

## Research Methodology

In this paper, the data were collected by paper questionnaire. Informed consent was given by each participant before experiments. The experiments were in accordance with the Declaration of Helsinki and approved by the Ethics Committee of the Department of Psychology, Peking University. Before the formal investigation, the research team carried out a trial investigation. The sample is mainly from three Chinese high-tech enterprises selected at random 215 questionnaires were sent out in the research phase, 215 of which were recovered and 213 were valid, with an effective recovery rate of 100%.

The formal survey sample is from high-tech enterprises in China, involving automation, information, and other industries. The research object is mainly the employees of high-tech enterprises, including ordinary employees, grass-roots managers, middle-level managers, and senior managers. The demographics of the official sample are shown in Table 1.

**TABLE 1:** List of effective sample composition of formal investigation (*N* = 213).

**Name**	**Category**	**Number of samples**	**Percentage**
Education	Junior College	38	17.8%
	Undergraduate course	107	52.2%
	Master’s degree	45	21.1%
	Dr.	18	8.5%
Work Nature	Production and technical services	129	60.6%
	R&D and Design	56	26.3%
	Researcher	12	5.6%
	Others	6	7.5%
Age	30 and below	30	37.6%
	31–40	95	44.6%
	41–50	30	14.1%
	51–60	8	3.8%
Gender	Female	53	24.9%
	Male	160	75.1%

Formal research has the following characteristics:

Sample selection. Mainly use the following three standards: First, According to the research objectives, this paper mainly focuses on the reality of China, so we select three high-tech enterprises in China to study. Secondly, the sample enterprise internal research object and research team have better social relations, to ensure a higher effective rate of questionnaire recovery. Thirdly, the type and scale of the enterprise. Because the object of this survey is innovative enterprises, the number of employees in such enterprises is generally more than 50, which can ensure that such enterprises have a complete management system. Measuring tools. In order to ensure the reliability and validity of measurement tools, this study was based on the existing literature use scale at home and abroad, combined with the actual situation of enterprises, and according to the survey results of the questionnaire items wording, number of appropriate revisions, so as to ensure the scientific and standardized measurement tools. Specific measurement tools are as follows:

According to “Gough (1979)’s Creative Personality Scale,” with 30 words in total (such as: original, talented, smart, etc.), the participants were asked to check the words that fit their own description.

Learning goal orientation, performance certification orientation and performance avoidance orientation were tested by Vande (1997), with 16 questions in total, such as: I’m willing to choose a challenging job that allows me to learn a lot, etc. In the study, “Likert’s 5-point scoring system” is used.

Employee innovation performance was measured by Zhou and George (2001). A total of 13 items, e.g., “I’ll come up with new ways to improve quality, etc.” were using Likert 5 points scoring. According to Zhou et al. (2008), the scale is more suitable for self-report measurement, which can capture the subtle and meticulous thinking and content of employees’ participation in the process of enterprise innovation more accurately and comprehensively. The results showed that the scale had good reliability and validity (= 0.972).

## Results

### Reliability and Validity Analysis – Homologous Variance Test

Because the questionnaires were all self-reported at the time of completion, homologous variance problem (CMV) may exist. The CMV of this study was detected by SPSS 25.0 using Harman’s single factor analysis. By factor analysis of the items in this study, three factors were separated out when they were not rotated. The strongest factor accounted for 44.19% of the total load and less than half of the total explanatory variance. Therefore, it can be concluded that there is no serious homologous variance problem in this study.

### Reliability and Validity Analysis – Validity Analysis of the Measurement Model

Using Amose 22.0 software, confirmatory factor analysis was performed on the three dimensions of the goal-oriented questionnaire. The fitting indexes of the model were as follows (see Table 2):

**TABLE 2:** Validity analysis results of the model.

	**Factor**	***x*^2^/df**	**RMSEA**	**CFI**	**AGFI**	**NFI**
Fitting standard		< 2	<0.08	>0.90	>0.90	> 0.90
Model	3	2.50	0.08	0.90	0.83	0.95
Fitting result		Reluctantly	Good	Good	Okay	Okay

From the matching index, it can be clearly seen that the confirmatory factor model of the scale matches the observed data well, which shows that the target-oriented three-dimensional division is appropriate. If the factor load of one item is less than 0.5 in Goal Oriented Questionnaire (GOQ), that is, the factor load of the other items is more than 0.5 and less than 0.95 after the fourth item of Performance Certificate Oriented Questionnaire (PQ4) is deleted, the factor load of the other items is more than 0.5 and less than 0.95. The combined reliability of the three dimensions was greater than 0.6, 0.98, 0.86, and 0.97, respectively, and the mean-variance extraction AVE was greater than 0.5, indicating good aggregation validity (see Table 3).

**TABLE 3:** Result of confirmatory factor analysis of variable aggregation validity.

	**Factor load**	**Reliability coefficient**	**Measurement error**	**Combined reliability**
Learning Objective Item 1	0.84	0.70	0.16	0.98
Learning Objective Item 2	0.91	0.83	0.09	
Learning Objective Item 3	0.97	0.94	0.03	
Learning Objective Item 4	0.94	0.88	0.06	
Learning Objective Item 5	0.93	0.87	0.07	
Learning Objective Item 6	0.85	0.73	0.15	
Performance Certificate Item 1	0.84	0.71	0.16	0.86
Performance Certificate Item 2	0.73	0.53	0.27	
Performance Certificate Item 3	0.61	0.37	0.39	
Performance Certificate Item 4	0.59	0.34	0.41	
Performance Avoidance Item 1	0.72	0.52	0.28	0.97
Performance Avoidance Item 2	0.87	0.75	0.14	
Performance Avoidance Item 3	0.96	0.93	0.04	
Performance Avoidance Item 4	0.95	0.91	0.05	
Performance Avoidance Item 5	0.87	0.75	0.13	

In addition, the square variance of the three dimensions was larger than the square of the correlation coefficient with the other two dimensions, and the discriminative validity of the questionnaire was good (see Table 4).

**TABLE 4:** Differential validity.

	**Learning goal orientation**	**Performance Certificate**	**Performance avoidance**
Learning goal orientation	0.9		
Performance certification orientation	0.72	0.61	
Performance avoidance orientation	−0.31	−0.26	0.89

### Analysis of the Influence of All Variables on Innovation Performance

The basic information of the subjects in this study is gender, age, education, nature of work, rank, and length of service. Age, length of service, educational level, job rank, and so on, are classified as ordered non-equal difference variables. If they are directly included in the model, the distance between different educational level, age, job rank, and so on is actually the same, which is obviously not in line with the actual situation. Gender is a classified variable and cannot be directly included in linear regression model. The variables of creative personality score, learning goal orientation, performance certification orientation and performance avoidance orientation are continuous variables, which can be directly incorporated into linear regression model. To sum up, we need to use the optimal scale regression. SPSS 25.0 was used. Through the optimal scale regression analysis, this paper analyzes the effect of each possible independent variable on innovation performance, and the data list is as follows: (see Table 5).

**TABLE 5:** Analysis of the influence of all variables on innovation performance.

**Analysis of the relationship between all variables and innovation performance**					
	**Normalization coefficient**		**Degree of freedom**	**F**	**Significance**
	**Beta**	**Self-service Sampling (1000) Estimation of Standard Errors**			
Gender	0.119	0.045	2	6.957	0.001
Age	0.259	0.098	1	6.955	0.009
Education level	0.150	0.065	2	5.398	0.006
Nature of work	−0.117	0.061	3	3.726	0.013
Job level	0.085	0.055	2	2.341	0.100
Length of service	−0.200	0.101	4	3.894	0.005
Creative personality Trait	0.084	0.045	1	3.431	0.066
Learning goal orientation	0.830	.	1	.	.
Performance certification orientation	−0.129	.	1	.	.
Performance avoidance orientation	−0.050	0.054	1	0.869	0.353

**Dependent variable: innovation performance**					
**A. Tolerance of this variable is less than 0.0001.**					
**Analysis of the relationship between all variables and innovation performance**					
	**Normalization coefficient**		**Degree of freedom**	**F**	**Significance**
	**Beta**	**Self-service Sampling (1000) Estimation of Standard Errors**			

Gender	0.119	0.045	2	6.957	0.001
Age	0.259	0.098	1	6.955	0.009
Education level	0.15	0.065	2	5.398	0.006
Nature of work	−0.117	0.061	3	3.726	0.013
Job level	0.085	0.055	2	2.341	0.1
Length of service	−0.2	0.101	4	3.894	0.005
Creative personality Trait	0.084	0.045	1	3.431	0.066

The data showed that when all variables were put into the regression model, the model fit was good overall, R was.74, and adjusted R was.71, *P* < 0.001. Job level, creative personality trait score and performance avoidance orientation had little influence on innovation performance (*P* > 0.05).

When only gender, age, education, nature of work, rank, and length of service were included in the regression model, the fit of the model is good, R was.37, adjusted R was.31, *P* < 0.001, that is to say, the variables of gender, age, education, job nature, job rank, and length of service had 31% contribution rate to employee innovation performance, and the variables of creative personality traits, learning goal orientation, performance certification orientation, and performance avoidance orientation had 40% contribution rate to employee innovation performance. In terms of gender, the standardized value is 0.58 for men and −1.72 for women, and the innovation performance of men is higher than that of women. In terms of age, the quantitative scores of 41–50, 51–60 years old are 1.99, and the quantitative scores of 31–40 and under 30 years old are −50. This indicates that the innovation performance of 41–60 years old is higher than that of under 40 years old. In terms of educational level, the innovation performance of Master and Doctor (both 1.085) is higher than that of Bachelor (−0.64), and Bachelor is higher than that of Bachelor (−1.785). In terms of the nature of the work, the researchers’ innovation performance was quantified as 2.1, higher than R & D and design personnel, Designers are quantified into.60, the designer is higher than the production and technical service personnel, the quantitative score of production and technical service is −0.81. From the job level, the quantitative score of innovation performance of senior and middle managers is 1.60, higher than that of grass-roots managers, the quantitative score of innovation performance of grass-roots managers is −0.45, and higher than that of ordinary employees, the quantitative score of innovation performance of ordinary employees is −0.71. In terms of the length of service, the quantitative score of innovation performance was 1.90 for 16 years and above, higher than that for 11–15 years (quantitative score.20). 11–15 years was higher than that for 8–10 years (quantitative score −0.52), 8–10 years was higher than that for 5–7 years, 2–4 years (all −0.75), and 0–1 year had the lowest quantitative score of −2.90.

### Analysis of the Relationship Among Creative Personality Traits, Goal Orientation, and Employees’ Innovation Performance

SPSS plug-in Sobel_spss. Sbs was used to analyze the mediating and moderating effects. The results showed that the total effect of creative personality traits on innovation performance was significant, with a total regression coefficient of 1.19 (*P* < 0.001). The direct effect of creative personality on innovation performance was 1.05; the direct effect of goal orientation on innovation performance was significant (0.72, *P* < 0.001). The mediating effect of learning goal orientation on creative personality traits and innovation performance was significant (0.73, *P* < 0.001). The mediating effect of performance certification orientation on creative personality traits and employees’ innovation performance was significant (0.73, *P* < 0.001). The mediating effect of performance certification orientation on creative personality traits and employee innovation performance was significant (−0.17, *P* < 0.01). In order to test the moderating effect of goal orientation on innovation personality and innovation performance, we use the interaction item (innovation personality × goal orientation) to judge the moderating effect. Amos 22.0 was used to test the moderating effect of goal orientation on creative personality traits and innovation performance. All the variables were significant, and OK: Default model appeared in the interface. The model and data were matched successfully. Therefore, the following conclusions are referential to some extent.

**FIGURE 1:**
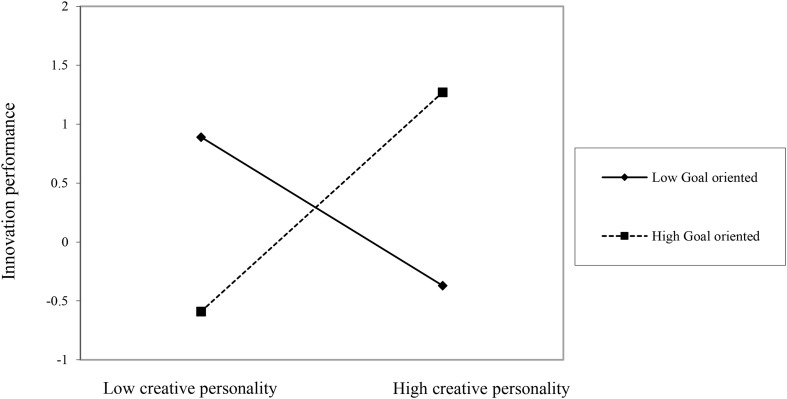
Goal orientation moderates the relationship between creative personality traits and employee innovation performance.

The results show that goal orientation moderates the relationship between creative personality traits and employee innovation performance (see Figure 1).

Employee innovation performance = 0.04 × goal-oriented + 0.78 × goal-oriented × creative personality trait + 0.15 × creative personality trait score, the significant level of interaction items <0.05. Therefore, this study shows that goal-oriented can significantly regulate the relationship between creative personality trait and innovation performance. Innovation Performance = (0.15 + 0.78 × Goal Orientation) Creative Personality Traits Score + 0.04 × Goal Orientation. The influence coefficient of creative personality traits on innovation performance is 0.15 + 0.78 × goal orientation. The significant critical value of T distribution and degree of freedom of 3 is 3.18, so when the target orientation is more than 2.36, the moderating effect is significant. 0 < goal orientation < 2.36, which means that goal orientation cannot significantly affect innovation performance. Because the coefficient (0.15 + 0.78 × goal orientation) cannot be less than 0, and goal orientation >2.36, goal orientation has a significant positive impact on employee innovation performance.

This paper analyzes the relationship among creative personality traits, learning goal orientation and innovation performance, and the mediating effect of learning goal orientation is significant. The total regression coefficient was 1.19, *P* < 0.001; The direct effect of creative personality traits on employees’ innovation performance was 0.47, and the direct effect of learning goal orientation on employees’ innovation performance was significant (=1.61, *P* < 0.001). The mediating effect of learning goal orientation on creative personality traits and innovation performance was significant (0.73, *P* < 0.001).

This paper analyzes the relationship among creative personality trait score, performance certification orientation and employee innovation performance, and the mediating effect of performance certification is significant. The total regression coefficient was 1.19, *P* < 0.001; the direct effect of creative personality traits on employees’ innovation performance was 0.47, and the direct effect of performance certification orientation on innovation performance was significant (=1.61, *P* < 0.001). The mediating effect of performance certification orientation on creative personality traits and employees’ innovation performance was significant (0.73, *P* < 0.001).

This paper analyzes the relationship among creative personality traits, performance avoidance orientation and employee innovation performance. The mediating effect of performance avoidance orientation is significant. Total regression coefficient = −0.52, *P* < 0.001; the direct effect of creative personality traits on employees’ innovation performance was -0.35, *P* < 0.01. The direct effect of performance avoidance orientation on employees’ innovation performance was significant (−0.14), *P* < 0.001. The mediating effect of performance certification orientation on creative personality traits and employee innovation performance was significant (−0.17, *P* < 0.01).

## Discussion

### Theoretical Significance

First of all, the conclusion of this study clearly points out the uniqueness of the internal mechanism of the impact of goal orientation on employee innovation performance in the Chinese context. Although the research has verified the impact of goal orientation on employee performance, the research is conducted in the western context (Kim et al., 2010). Some scholars point out that Chinese traditional culture pays attention to “moderation” and “harmony,” and employees tend to obey and follow the crowd in the workplace. These cultural factors will profoundly affect the performance of employees’ creative personality traits, and may draw different conclusions from the western situation (Zhou and Hoever, 2014). This paper examines the role of goal orientation in promoting employee innovation performance in the context of China. The results show that goal orientation has a certain cross-cultural universality in promoting employee innovation performance, and goal orientation research has a certain reference and guiding significance.

Secondly, previous researches on employee innovation performance at the micro-level mostly focused on how managers actively promote and stimulate employee innovation performance. This kind of research is still based on the traditional hypothesis that employees are relatively passive objects in the process of enterprise innovation and need external factors to stimulate their innovation motivation. However, in recent years, many studies have shown that employees in the workplace and organizational context are goal-oriented subjects, rather than simply passive, reactive objects (Zhou and Hoever, 2014). To this end, it is pointed out that future research should start from the self-management behavior of employees, in order to stimulate the ability of employees, break through the innovation dilemma to provide an effective new way (Stobbeleir et al., 2011). Starting with goal orientation, this paper assumes and verifies the positive relationship between learning goal orientation, performance certification orientation and innovation performance, and the negative relationship between performance avoidance goal orientation and innovation performance. Therefore, based on the goal-oriented perspective, using a sample of high-tech enterprises in China, this paper brings creative personality, goal-oriented and employee innovation performance into the scope of the study, to explore the antecedents and impact mechanism of employee innovation performance, which is a complement and expansion to the existing research in the field of employee innovation performance.

Thirdly, this paper explores the influence mechanism of creative personality traits on employees’ innovation performance, opens the “black box” of personality traits to employees’ innovation performance, and verifies the moderating effect of goal orientation on the relationship between employees’ creative personality and employees’ innovation performance. Therefore, this paper takes the creative personality traits of enterprise employees as the research object, analyzes the moderating effect of goal orientation, and reveals the relationship between creative personality traits, goal orientation, and employee innovation performance, which is a further expansion of the existing research.

Fourthly, this study shows that creative personality traits have a significant positive effect on employees’ innovation performance, it is aimed at the creative personality traits of enterprise employees. But, existing domestic studies tend to focus on the relationship between adolescents’ or college students’ creative personality traits and their creative ability or creativity. This is a further expansion of the existing research.

Finally, this study shows that the mediating effects of learning goal orientation, performance certification orientation and performance avoidance orientation are all significant in the creative personality traits and employee innovation performance. Previous studies focused on the relationship between “Big Five personality,” goal orientation and behavioral performance. This study examined the relationship between specific creative personality traits and goal orientation in response to Paunonen and Jackson (2000). At the same time, this study reveals the positive effects of learning goal orientation and performance certification orientation on employees’ innovation performance. Previous studies agree that learning goal orientation plays an important role in innovation performance, but there is no consistent conclusion on the relationship between performance certification orientation and employee innovation performance. This study shows that learning goal orientation and performance certification orientation are associated with stimulating employee innovation performance. This study further enriches the research on the relationship between goal orientation and employee innovation performance.

### Practical Significance

This study not only helps to understand employee innovation behavior from the perspective of goal orientation, but also provides a new perspective for exploring employee innovation management in practice in the future. At present, enterprises mainly emphasize on stimulating employees’ innovation motivation by improving the external environment and giving material incentives (Zhang et al., 2014), but ignore the goal-oriented self-management mechanism. Under the circumstances of increasingly fierce external competition, complicated internal situation and highly awakened employees’ self-consciousness, it is often difficult for the formal management mechanism of enterprise organizations to manage, motivate and restrain the employees’ work objectives and work contents. At this time, whether the enterprise can stimulate and adjust employees’ self-management consciousness and goal orientation through a series of measures will play a more and more important role in improving human resources and achieving sustainable growth.

Specifically, the practical significance of this study mainly includes as follows:

First of all, enterprises should pay attention to the role of employee creative personality traits, enterprises can use creative personality scale and other measurement tools to identify the creative personality traits of newly elected employees.

Secondly, enterprises should fully consider and identify candidates’ learning goal orientation, performance certification orientation, performance avoidance orientation, and other goal orientation.

Finally, when the enterprise manages the existing employees, on the one hand, it should advocate the improvement of employees’ learning skills, create an environment conducive to employees’ learning and exploring new knowledge to strengthen employees’ learning goal orientation, and provide more knowledge acquisition channels for learning goal-oriented employees by strengthening training, to help them generate useful new ideas. At the same time, through encouraging employees to show their abilities, setting up reasonable innovation performance targets and establishing corresponding incentive system, and rewarding the employees who have outstanding innovation performance, the performance-oriented employees focus on the enterprise innovation work in order to prove their abilities. In addition, enterprises can also recognize some employees with high innovation performance as innovation examples in the organization, constantly promote mutual learning among colleagues and cultivate employees’ ability to imitate the will of employees, improve the overall innovation performance of employees, in order to help enterprises to create and improve the innovation path.

### Limitations and Avenues for Future Research

First of all, in studying employees of high-tech enterprises, we recognize that such employees generally show high levels of professional initiative, strong capacities for innovation, and a high degree of self-regulation and self-management. Further, in being based on cross-sectional data, the results of the study may not reflect dynamic causality between variables. Future studies may be performed over several stages and examine different industries and enterprises to draw more generalizable conclusions.

Secondly, in studying employees’ innovation performance from the perspective of goal orientation. We measured learning, attestation, and avoidance according to goal orientation theory. Future work can further expand on our research mechanisms and variables and explore the influence of other forms of proactive self-management behavior (e.g., advising, active socialization, etc.) on employees’ innovation performance.

Thirdly, this study only focused on individual differences and did not consider the impact of organizational contexts on creative personality traits. Future studies may also consider situational variables such as leadership and organizational climates.

## Data Availability Statement

The original contributions presented in the study are included in the article/supplementary material, further inquiries can be directed to the corresponding author.

## Author Contributions

KZ conceptualized the manuscript and wrote the complete draft.

## Conflict of Interest

The author declares that the research was conducted in the absence of any commercial or financial relationships that could be construed as a potential conflict of interest.
